# Theoretical assessment data for the binding of sepsis causing pathogens to ApoH beads

**DOI:** 10.1016/j.dib.2017.04.052

**Published:** 2017-05-05

**Authors:** Manjula Ramya Vutukuru, Nivedita Mitra

**Affiliations:** Technology Center, Siemens Healthcare Private Limited, Bengaluru 560100, India

## Abstract

The data presented in this paper supports the research article “A rapid, highly sensitive and culture-free detection of pathogens from blood by positive enrichment” ( Vutukuru et al., 2016) [1]. We compared a list of sepsis causing pathogens to the ApoH binding data given to us by ApoH technologies. The data highlights the binding of ApoH beads to sepsis causing pathogens.

## **Specifications Table**

**Table:** 

Subject area	*Biology*
More specific subject area	*Molecular Diagnostics*
Type of data	*Table*
How data was acquired	*Theoretical assessment*
Data format	*Analyzed*
Experimental factors	*List of sepsis causing pathogens is obtained from the literature and the list of ApoH binding to pathogens is obtained from ApoH technologies web site. This information is based on experimental studies conducted by ApoH technologies.*
Experimental features	*An assessment was done to look at the % of sepsis causing pathogens that bind to ApoH beads, to determine the universality of the ApoH enrichment technology.*
Data source location	*The data sources are listed in the References.*
Data accessibility	*Within the data in brief article*

## **Value of the data**

• The data demonstrates the value of using ApoH beads for pathogen enrichment in the original paper [1].
• The data presented here will allow other researches to replicate these experiments and allow them to use the tools we used here in other similar contexts.

## 1 Data

The data presented in Table 2 supports the research article **“**A rapid, highly sensitive and culture-free detection of pathogens from blood by positive enrichment” [1].

Table 1 shows a list of primers and probes used in the study [2], [3], [4]. These primers and probes have been used in qPCR experiments to quantify the pathogens from whole blood, after ApoH bead binding.

**Table 1:** Primers and probes used in the study. Numbers in square brackets indicate the references from where the sequence was taken.

**Primer/Probe name**	**Sequence**
*E. coli* 16S rDNA – Forward [2]	5′ – CCAGACTCCTACGGGAGGCAG – 3′
*E. coli* 16S rDNA – Reverse [2]	5′ – CGTATTACCGCGGCTGCTG – 3′
*E. gallinarum* 16S rDNA – Forward [2]	5′ – CCAGACTCCTACGGGAGGCAG – 3′
*E. gallinarum* 16S rDNA – Reverse [2]	5′ – CGTATTACCGCGGCTGCTG – 3′
*C.tropicalis* 18S rDNA – Forward [3]	5′ – CATTGCGCCCTTTGGTATTC – 3′
*C.tropicalis* 18S rDNA – Reverse [3]	5′ – GTTGAGCAATACGCTAGGTTTG – 3′
*E. coli* tna rRNA – Forward [4], 126 bp amplicon	5′ – GGGGCGGTGACGCAG– 3′
*E. coli* tna rRNA – Forward [4], 372 bp amplicon	5′ – CATTACCATTCGTGTTATTG– 3′
*E. coli* tna rRNA – Reverse [4], 126 bp and 372 bp amplicon	5′ –CCTGGTGAGTCGGAATGGTG– 3′
*E. coli* V3 Taqman Probe [2]	VIC – 5′ – TTGACGTTACCCGCAGAAGAAGCA – 3′–BHQ
*E. gallinarum* V3 Taqman Probe [2]	FAM – 5′ – TGGACGAAAGTCTGACCGAGCAAC– 3′–BHQ
*C.tropicalis* Taqman Probe [3]	JOE– 5′ – TGAGCGTCATTTCTCCCTCAAACCC– 3′–BHQ
*E. coli* tna Taqman Probe [4]	CY5– 5′ – CGATGATGCGCGGCG– 3′–BHQ

Table 2 shows a list of sepsis causing pathogen [5] and their respective prevalence. The pathogens highlighted in yellow have been shown to bind with ApoH beads as listed in the ApoH technologies website (http://www.apohtech.com/index.php?id=2).

**Table 2:** Suspected primary microbiologic pathogens in septic shock [5] and their binding to ApoH beads from the ApoH-CaptoBAC kit.
